# Recurrence of hepatitis C virus genotype-4 infection following orthotopic liver transplantation: Natural history and predictors of outcome

**DOI:** 10.4103/0256-4947.51796

**Published:** 2009

**Authors:** Hatim Mudawi, Ahmed Helmy, Yasser Kamel, Mohammed Al Saghier, Mohammed Al Sofayan, Mohammed Al Sebayel, Hatem Khalaf, Hamad Al Bahili, Yasser Al Shiek, Khalil Alawi, Ahmed AlJedai, Hazem Mohamed, Waleed Al Hamoudi, Ayman Abdo

**Affiliations:** a Department of Liver Transplantation, King Faisal Specialist Hospital and Research Centre, Riyadh, Saudi Arabia; b Division of Pharmacy, King Faisal Specialist Hospital and Research Centre, Riyadh, Saudi Arabia; c College of Medicine, King Saud University, Riyadh, Saudi Arabia

## Abstract

**BACKGROUND AND OBJECTIVES:**

There are few reports on hepatitis C virus genotype 4 (HCV-4) recurrences after orthotopic liver transplantation (OLT). Therefore, we undertook a study to determine the epidemiological, clinical and virological characteristics of patients with biopsy-proven recurrent HCV infection and analyzed the factors that influence recurrent disease severity. We also compared disease recurrence and outcomes between HCV-4 and other genotypes.

**PATIENTS AND METHODS:**

All patients who underwent OLT (locally or abroad) for HCV related hepatic cirrhosis from 1991 to 2006 and had recurrent HCV infection were identified. Clinical, laboratory and pathological data before and after OLT were collected and analyzed.

**RESULTS:**

Of 116 patients who underwent OLT for hepatitis C, 46 (39.7%) patients satisfied the criteria of recurrent hepatitis C. Twenty-nine (63%) patients were infected with HCV genotype 4. Mean (SD) for age was 54.9 (10.9) years. Nineteen of the HCV genotype 4 patients (65.5%) were males, 21 (72.4%) received deceased donor grafts, and 7 (24.1%) developed ≥1 acute rejection episodes. Pathologically, 7 (24.1%) and 4 (13.8%) patients had inflammation grade 3-4 and fibrosis stage 3-4, respectively. Follow-up biopsy in 9 (31%) HCV genotype 4 patients showed stable, worse and improved fibrosis stage in 5, 2 and 2 patients, respectively. Of the 7 patients in the recurrent HCV group who died, 6 were infected with genotype 4 and 4 of them died of HCV-related disease.

**CONCLUSION:**

This analysis suggests that HCV recurrence following OLT in HCV-4 patients is not significantly different from its recurrence for other genotypes.

End-stage liver disease secondary to hepatitis C virus (HCV) infection is the major indication for orthotopic liver transplantation (OLT) worldwide.^1^ Re-infection of the graft is universal and occurs shortly after transplantation, leading to an accelerated course of liver injury in many cases.^2^ Most studies conducted worldwide have investigated disease recurrence in HCV genotypes 1, 2, and 3.^1^ However, there are very few reports of post-OLT recurrence of HCV genotype 4 (HCV-4), the predominant genotype in the Middle East.^3^–^6^ Therefore, the aims of this retrospective study were to first, describe the epidemiological, clinical and virological characteristics of all patients diagnosed with biopsy-proven recurrent HCV-4 who were followed at King Faisal Specialist Hospital and Research Center (KFSHRC) in Riyadh, Saudi Arabia; second, determine the host, viral and donor variables that may influence severity of disease recurrence; and finally, compare genotype 4 with other genotypes in terms of epidemiological, clinical and pathological characteristics in addition to disease outcome.

## PATIENTS AND METHODS

All patients who received OLT (whether locally or abroad) for liver disease secondary to HCV infection and were followed at King Faisal Specialist Hospital and Research Centre (KFSHRC), Riyadh, Saudi Arabia, from 1991 through the end of 2006 were identified through a computerized database. The study protocol was approved by the department and the local ethical and research committees. The patient charts, transplant information, and laboratory data were reviewed. Only patients with recurrent HCV infection were included in the analysis. Recurrent hepatitis C was defined as: 1) alanine aminotransferase (ALT) or aspartate aminotransferase (AST) ≥ 2 times the upper limit of normal; 2) positive HCV RNA polymerase chain reaction (PCR); and 3) liver biopsy consistent with recurrent hepatitis C, with no evidence of graft rejection. Patients were excluded from the study if they had liver biopsy evidence of acute rejection, biliary obstruction, ischemic hepatitis, or any other viral infections. Demographic data, including age and gender of the recipient, type of OLT, donor age, presence and number of acute cellular rejection episodes, viral load pre- and post-transplant, HCV genotype and primary immunosuppressive therapy, were all collected. All patients were followed-up in the post-liver transplant clinic at least every 2 months based on their condition, with regular clinical, biochemical, imaging and virological examinations as appropriate, in addition to the pathological assessment.

HCV detection and quantification were performed using the Abbott Real-Time M2000rt instrument. The HCV assay detects and quantifies genotypes,^1^–^6^ and the Abbott Real-Time HCV assay provides detection limits (analytical measurement range) from 30 to 100 000 000 IU/mL, where 1 IU/mL=4 copies/mL.

Histopathological assessment was performed according to the METAVIR scoring system by a single liver histopathologist.^7^ In our center, protocol liver biopsies are not practiced and the decision to perform liver biopsy is made if the serum ALT and/or AST levels are ≥ 2 times the upper limit of normal, or there is a consistent increase in direct bilirubin. The normal reference range of serum ALT and AST in our laboratory is 10-45 IU/L.

Data was collected initially on a specialized data collection form, imported into a Microsoft Excel work-sheet and finally transferred to the statistical package for social sciences (SPSS) version 11.0 (SPSS, Chicago, IL, USA) for analysis. Means of continuous variables were compared using the *t* test, non-parametric tests (Wilcoxon and Mann Whitney) or one-way analysis of variance (ANOVA), in addition to the post-hoc test (Tukey) as appropriate. The chi-square or Fisher exact tests were used to compare frequencies and proportions. Multivariate stepwise logistic regression analysis was performed to determine the independent predictors of fibrosis. A *P* value <.05 was considered statistically significant. Cumulative survival was calculated by the life-table method (Kaplan-Meier analysis) from the date of transplantation until the last follow-up, death, or loss of follow-up. Survival of patients in different groups was compared by the log-rank test.

## RESULTS

Of the 116 patients who received transplants due to hepatitis C and were followed in our center, 46 satisfied all three inclusion criteria for having recurrent HCV infection post-OLT. Of these 46, 33 (71.7%) were males and the mean (SD) for age was 54.5 (11.2) years. Thirty (65.2%) patients received deceased donor grafts, while 16 (34.8%) received living-related donor grafts. The mean (SD) for donor age was 29.5 (9.8) years and 86% of donors were under the age of 40 years. Twenty-seven patients (58.7%) received transplants at KFSHRC, with the remaining 19 patients receiving transplants abroad, mostly in China, the USA and Germany. Nine (19.9%) patients developed ≥1 episode of acute rejection during follow-up. Clinical, biochemical and virological characteristics of the patients included in our study are shown in Table 1.

**Table 1:** Clinical, biochemical and virological characteristics of patients with hepatitis C virus recurrence post-liver transplantation atthe time of diagnosis (n=46).

Variable	Mean (SD) or n(%)
Mean recipient age (years)	54.5 (11.2)

Mean donor age (years)	29.5 (9.8)

Sex	
Male	33 (71.7)
Female	13 (28.3)

Type of transplant	
Deceased donor	30 (65.2)
Living donor	16 (34.8)

Transplants site	
KFSH&RC	27 (58.7)
Abroad	19 (41.3)

Rejection	
yes	9 (19.9)
No	37 (80.1)

Intravenous pulse steroids	
yes	3 (6.8)
No	43 (93.2)

Mean period on steroids (months)	10.9 (14.3)
Survived	
yes	38 (82.2)
No	8 (17.8)

Immunosuppression at biopsy time	

Single drug	14 (30.4)
Combination	32 (69.8)

Transplant–biopsy period (days)	687.8 (1094.1)

Positive PCR attime of transplant	45 (97.8)

HCV genotype	
4	29 (63)
Others	17 (37)

Viral load at transplant (million copies/mL)	2.4 (1.8)

Viral load at biopsy (million copies/mL)	1.8 (1.7)

ALT at biopsy (IU/L)	206 (174.3)

AST at biopsy (IU/L)	205.5 (244.2)

ALT: alanine aminotransferase. AST: aspartate aminotransferase. n: number. HCV: hepatitis C virus. KFSHRC: King Faisal Specialist Hospital and Research Centre.

At the time of graft biopsy, 14 (30.4%) patients were treated with either tacrolimus or cyclosporine alone, while the remaining 32 (69.6%) received a combination of drugs, mostly either cyclosporine and mycophenolate mofetil (MMF) or tacrolimus and MMF Table 1. Twenty-six (56.5%) patients were taking steroids at the time of biopsy, and only three (6.5%) received IV steroids for treatment of acute rejection episodes. None of our patients received OKT3 or any other anti-lymphocyte preparation.

Upon initial histological examination, 5 (10.9%) patients had grade 0 inflammation, 10 (21.9%) had grade 1, 21 (45.7%) had grade 2, 8 (17.4%) had grade 3 and 2 (4.3%) had grade 4 inflammation. Fibrosis scoring was performed and showed that 14 (30.4%) patients had fibrosis stage 0, 17 (37.0%) stage 1, 10 (21.7%) were stage 2, 4 (8.7%) were stage 3, and only 1 (2.2%) was stage 4 (cirrhosis). The mean (SD) time between transplant and biopsy was 687.8 (1094.1) days. Seventeen (36%) patients had at least one repeat liver biopsy during the follow-up. Six patients showed worsening fibrosis scores, eight were stable and three improved.

Patients who received OLT at KFSHRC (n=27) were similar to those who had OLT abroad (n=19) in demographic, clinical, biochemical, and virological variables. No significant differences were found between patients who received grafts from living-related donors (n=16) compared with patients who received deceased-donor grafts (n=30) in demographic, clinical, biochemical and virological variables except for HCV viral load at the time of biopsy, which was significantly higher in patients who received deceased donor grafts (*P*=.008).

Twenty-nine patients (63.0%) began peginterferon alfa-2a (40 KD; Pegasys, F. Hoffmann-La Roche, Basel, Switzerland) 135-180 mcg per week subcutaneously and ribavirin (Copegus, F. Hoffmann-La Roche, 800-1200 mg per day orally) therapy for 48 weeks based on their initial histological scores (patients were treated if they had elevated ALT of more than 2 times the upper limit of normal and had grade 2 or more inflammation and stage 2 or more fibrosis on liver biopsy. Of these, 16 (55%) were negative for HCV RNA at the end of therapy and three of these 16 (18.8%) relapsed after the end of treatment, resulting in a sustained virological response of 44.8% (13 of 29). After a mean (SD) follow-up of 38.8 (40.8) months (range, 7.9 to 173 months), 4 patients (in addition to the 1 patient who had cirrhosis at the initial liver biopsy) developed cirrhosis; 7 (15.5%) eventually died of liver failure; 1 died of unrelated causes; and the remainder of the patients were still alive at the time of writing.

Of the 46 patients who had recurrent hepatitis C, 29 (63.0%) were infected with genotype 4. Mean (SD) for age was 54.9 (10.9), 19 (65.5%) were males, 21 (72.4%) received deceased donor grafts and 7 (24.1%) developed ≥1 acute rejection episodes. Upon histological examination of liver biopsy samples from these genotype 4 patients (n=29), 4 (13.8%) patients had grade 0 inflammation, 6 (20.7%) had grade 1, 12 (41.4%) had grade 2, 5 (17.2%) had grade 3, and 2 (6.9%) had grade 4 inflammation. Fibrosis scoring showed that 11 (37.9%) patients had fibrosis stage 0, 9 (31.0%) stage 1, 5 (17.2%) were stage 2, 3 (10.3%) were stage 3, and only 1 (3.4%) was stage 4 (cirrhosis). Univariate analysis showed that there was a significant difference between recurrent HCV patients with fibrosis stage ≥2 compared to those with fibrosis stage 0-1 in the viral load at the time of biopsy (*P*=.04) (Table 2). In addition, there were significant differences in the transplantation-to-biopsy interval (*P*=.002) and the activity (inflammatory) grade (*P*=.008). However, multivariate logistic regression analysis of these three variables showed that only the viral load at the time of biopsy was an independent predictor of higher fibrosis stage in patients with HCV-4 recurrence post-OLT (*P*=.029).

**Table 2:** Univariable predictors of hepatic fibrosis in patients with recurrent HCV post-liver transplantation (n=46).

Variable	Fibrosis stage 0-1 (n=31)	Fibrosis stage 2-4 (n= 15)	*P*
Sex (M/F)	23 (74.2)/8 (25.8)	10 (66.7)/5 (33.3)	.60
Mean recipient age (≤40/>40y)	3 (9.7)/28 (90.3)	1 (6.7)/14 (93.3)	.73
Rejection (no/yes)	25 (80.6)/6 (19.4)	12 (80.0)/3 (20.0)	.96
Steatosis in biopsy (no/yes)	20 (64.5)/11 (35.5)	8 (53.3)/7 (46.7)	.47
Mean viral load (low/high)	11 (35.5)/20 (64.5)	1 (6.7)/14 (93.3)	.037*
Transplant type (DDLT/LDLT)	22 (71)/9 (29)	8 (26.7)/7 (46.7)	.24

Transplant site (abroad/KFSHRC)	14 (45.2)/17 (54.8)	5 (33.3)/10 (66.7)	.45
Viral genotype (4/others)	20 (69)/9 (31)	9 (69.2)/4 (30.8)	.99
Steroids (no/yes)	6 (20.7)/23 (79.3)	12 (80.0)/3 (20)	.0001*
MMF (no/yes)	11 (37.9)/18 (62.1)	13 (86.7)/2 (13.3)	.002*

Data are expressed as mean±SD or n (%) as appropriate. MMF: mycophenolate mofitel. LDLT: living donor liver transplantation. DDLT: deceased donor liver transplantation. KFSHRC: King Faisal Specialist Hospital and Research Centre. Viral load low=<40 000 000 copy/mL; high=>40 000 000 copy/mL.

There was no significant difference between patients with HCV-4 and those infected with other genotypes in virological and histological parameters (Table 3). More patients (65.6%) with HCV-4 had hypertension than patients infected with other genotypes (30.8%, *P*=.036). Seventeen (48%) patients received pegylated interferon and ribavirin therapy, nine of whom responded to therapy and two relapsed after the end of the treatment response, resulting in a sustained virological response of 41%. During 38.5 months of follow-up (range, 7.9-170.5 months), 9 patients had a repeat liver biopsy, 5 showed a stable fibrosis score, 2 showed worsening and 2 showed improvement.

**Table 3:** Comparison of HCV genotype 4 and other genotypes in 46 liver transplant recipients with recurrent HCV infection.

Variable	Genotype 4 (n=29)	Other genotypes (n=17)	*P*
Sex (M/F)	19 (65.5)/10 (34.5)	14 (82.4)/3 (17.6)	.21
Mean (SD) age at transplant (years)	54.9 (10.9)	51.7 (12.9)	.41
Donor mean (SD) age (years)	30.6 (10)	25.3 (6.5)	.21
Survival at 12/36 months (%)	88.8/73.6	100/86.7	.30
Death	6 (20.7)	2 (11.8)	.30
Viral load pre-transplant (rank)*	9.9	11.6	.59
Viral load at time of biopsy (rank)*	22.5	19.4	.45
ALT attime of biopsy (IU/L)	179 (148.4)	246 (219.9)	.28
AST at time of biopsy (IU/L)	175 (181.9)	278.7 (257.6)	.54
Mean transplant-biopsy period (days)	786 (1148.3)	532 (1157.1)	.53
Rejection	9 (31.0)	3 (17.6)	.60
Grade 2-4 inflammation	19 (65.5)	12 (70.6)	.46
Stage 2-4 fibroses	9 (31)	6 (35.3)	.99
Diabetes mellitus	17 (58.6)	12 (70.6)	.86
Hypertension	19 (65.5)	6 (35.3)	.04
Hyperlipidemia	1 (3.4)	1 (5.9)	.55

Data are expressed as mean±SD or n (%) as appropriate. SD: standard deviation. ALT: alanine aminotransferase. AST: aspartate aminotransferase. n: number.

* Mean rank as calculated by Mann-Whitney test

Eight of the 46 patients (17.4%) died during the follow-up. Of those, 6 were infected with HCV-4; of these, 4 died of severe recurrent cholestatic hepatitis C or chronic graft failure secondary to hepatitis C. No significant difference was found between genotype 4 and non-genotype-4 patients in terms of survival during the follow-up period (Figure 1a). Similarly, survival analyses using Kaplan-Meier curves and the log-rank test were similar in the patients who had DDLT and LDLT as well as among those who had OLT locally and abroad (Figure 1b and 1c).

**Figure 1a:**
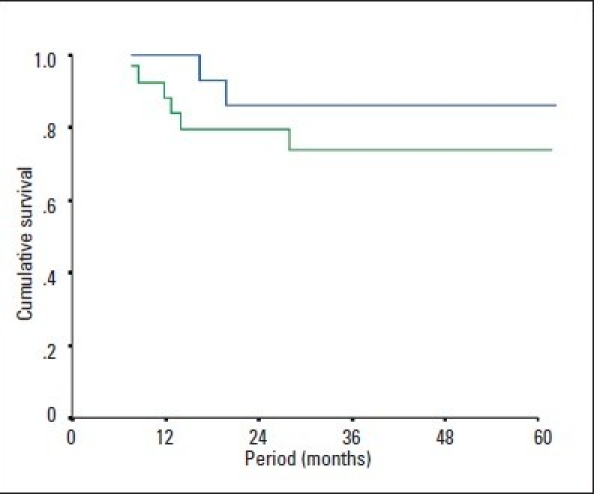
Kaplan-Meier survival analysis in genotype-4 patients (green) versus those with other genotypes (blue), *P*=.297 by logrank test.

**Figure 1b:**
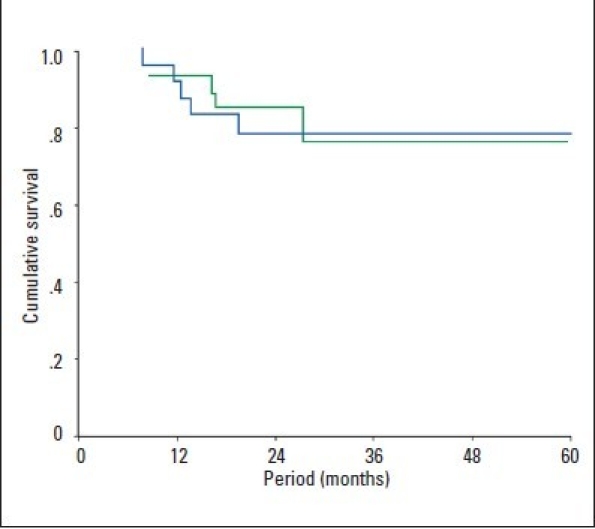
Kaplan-Meier survival analysis in patients who received transplantation locally (blue) versus those who were transplanted abroad (green), *P*=.869 by log-rank test.

**Figure 1c:**
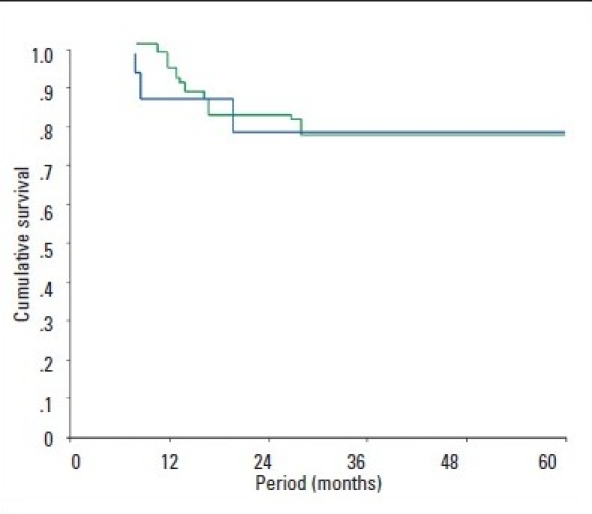
Kaplan-Meier survival analysis in patients who received DDLt (blue) versus those who received LDLt (green), *P*= .778 by log-rank test.

## DISCUSSION

Liver disease secondary to HCV infection is the most common indication for OLT worldwide, as well as in Saudi Arabia.^1^,^8^ Whether receiving the transplant locally or abroad, the majority of Saudi patients who receive transplants due to hepatitis C are infected with HCV-4.^9^,^10^ Although HCV genotype has not been shown to affect the natural history of chronic hepatitis C,^11^ the effect of genotype following OLT is controversial.^12^–^14^ There are only a few reports describing the natural history of recurrent HCV-4 after OLT in patients infected with genotype 4, most of which are conflicting.^3^–^6^ Gane et al reported on 14 patients with recurrent HCV-4 post-OLT and found that about 50% of these patients have progressive liver disease.^4^ Similarly, an analysis of 182 patients transplanted for HCV in Australia and New Zealand (16 of whom had HCV-4) found that, among many factors studied in a univariate and multivariate analyses, genotype 4 was associated with an increased risk for re-transplantation and death.^5^ In contrast, a study from another Australian center, including patients with HCV-4, showed that genotype 1b, not 4, was associated with higher recurrence rates after transplant.^6^ In a more detailed study from the UK, 32 of 128 patients who underwent OLT for hepatitis C were infected with HCV-4. A statistically significant greater fibrosis progression rate was observed in HCV-4 patients compared to non-genotype-4 ones, although their rates of survival were similar.^3^ The authors attributed the difference between these two groups to the significantly older donor age in the HCV-4 group and the ethnic background of these patients (predominantly Egyptian). They stated that: “donor livers that were suitable for transplantation but unsuitable or not needed for citizens of the United Kingdom (UK) were offered to non-UK citizens who formed a second waiting list.”^3^ This policy may have led to the selection of inferior grafts for HCV-4 patients, who are predominantly non-UK citizens, leading to inferior results in these patients. When reporting the histological findings, these investigators used the last available scored biopsy (showing significantly higher fibrosis progression rates). However, they did not report the findings of the initial liver biopsy, which was done according to a day-six protocol. It would have been interesting to see if indeed the quality of grafts given to patients with HCV-4 was inferior to that of those given to other patients.

In the current study, we report the results of patients who have biopsy-proven recurrent hepatitis C infection and make comparisons between patients with HCV-4 and non-HCV-4 genotype. Contrary to previous reports, our study showed that there were no significant differences between these two groups in terms of epidemiological, clinical and histological factors and outcome. We found that in the initial liver biopsy, which was performed after a mean time from transplantation of more than 2 years, there were only four patients who had fibrosis scores greater than stage 3. Two of these patients had worsening fibrosis on subsequent biopsies.

Although the majority of deaths in our study were in genotype-4 patients (6 of 8 deaths), there were only 4 hepatitis C virus-related deaths in the HCV-4-infected patients (13% mortality) after a mean (SD) of 14.7 (6.6) months of follow-up and we found no statistical difference in survival between genotype-4 patients and patients infected with other genotypes. The relatively good results seen in our HCV-4 patients (44.8% sustained viral response) may be explained by a number of factors. First, donor selection tends to be extremely conservative in our center, as marginal livers are very frequently rejected. Second, our mean donor age (about 30 years), an important parameter in post-transplant outcome, especially in hepatitis C patients, is significantly lower than what has been described in other studies.^15^,^16^ Third, only 6.8% of patients used IV steroids, and none used anti-lymphocyte preparations. Fourth, the HCV mean viral load at the time of transplant seems to be lower in our study compared to previously reported studies on the same genotype. Fifth, we based this analysis on the initial liver biopsy with the first elevation of liver enzymes, although the mean time from transplantation to initial biopsy was more than 650 days. Among many factors included in our analyses, the only factor predictive of an advanced histological score was the HCV RNA level at the time of biopsy. Although some studies have shown a positive correlation between pre-transplant viral load and severity of HCV recurrence after trans-plantation,^17^,^18^ others have not shown a consistent correlation between HCV viral load post-liver transplant and histological outcome.^12^,^13^ Our data suggest that this correlation may be important in at least genotype-4 patients. This is also supported by the fact that all patients who received antiviral therapy and responded to it had either stable or improved histological scores. Recently, treatment of hepatitis C post-OLT has been shown to improve survival in such patients, further suggesting a correlation between viral load and severity of disease.^19^

The main strength of the present study is that it included patients who had transplantation locally in our center as well as patients who received transplantations abroad. In addition, it included patients who received deceased donor and living related grafts. It also included patients with genotype 4 of an ethnic background different from the one described in most previous studies reporting on genotype-4 patients (Saudi and Egyptian patients). All these factors gave a wider variety of geno-type-4 patients, which allows study of this genotype in a more comprehensive fashion. Additionally, this study had a long follow-up time.

The outcome of recurrent HCV-4 appears to be not affected by the type of liver OLT (LDLT or DDLT). This may emphasize a greater role for host, viral, donor and therapeutic variables on disease severity. Although this is an issue that generated some debate recently, a recent study by Lawal et al^20^ showed no difference in the cumulative incidence of histological recurrence of HCV post-OLT or in the survival between recipients of DDLT (n=32) and those with split liver transplants (n=17), which is consistent with our results. The same result was also shown by a retrospective study involving 289 HCV patients, which concluded that LDLT does not increase HCV recurrence and progression.^21^ However, both studies^20^,^21^ came from areas where geno-type 1 infection predominates. Further studies are needed to assess the role of transplant type on the severity of recurrent HCV-4.

Our study suffers from all the weaknesses inherent to a retrospective analysis. In addition, because we have not been practicing protocol biopsy in our center, it was extremely hard to draw strong conclusions from the fibrosis progression results we have reported. In conclusion, our study suggests that HCV recurrence post-OLT in genotype-4 patients does not seem significantly different from recurrence in other genotypes.
